# A real-time epilepsy seizure detection approach based on EEG using short-time Fourier transform and Google-Net convolutional neural network

**DOI:** 10.1016/j.heliyon.2024.e31827

**Published:** 2024-05-23

**Authors:** Mingkan Shen, Fuwen Yang, Peng Wen, Bo Song, Yan Li

**Affiliations:** a School of Engineering, University of Southern Queensland, Toowoomba, Australia; b School of Engineering and Built Environment, Griffith University, Gold Coast, Australia; c School of Mathematics, Physics and Computing, University of Southern Queensland, Toowoomba, Australia

**Keywords:** Epilepsy seizure detection, EEG, Real-time, STFT, Google-net CNN

## Abstract

Epilepsy is one of the most common brain disorders, and seizures of epilepsy have severe adverse effects on patients. Real-time epilepsy seizure detection using electroencephalography (EEG) signals is an important research area aimed at improving the diagnosis and treatment of epilepsy. This paper proposed a real-time approach based on EEG signal for detecting epilepsy seizures using the STFT and Google-net convolutional neural network (CNN). The CHB-MIT database was used to evaluate the performance, and received the results of 97.74 % in accuracy, 98.90 % in sensitivity, 1.94 % in false positive rate. Additionally, the proposed method was implemented in a real-time manner using the sliding window technique. The processing time of the proposed method just 0.02 s for every 2-s EEG episode and achieved average 9.85- second delay in each seizure onset.

## 1 Introduction

Epilepsy is a neurological disorder characterized by abnormal synchronous electrical activity in the brain [1]. It affects a significant number of people worldwide, with an estimated 50 million individuals living with epilepsy [2]. Given the high prevalence of epilepsy and the impact of seizures on patients, there is a growing need to improve the efficiency of diagnosis and treatment. One approach to address this is to develop real-time automatic detection systems using electroencephalogram (EEG) signals [3].

The main features of EEG seizure active signals are the spikes waves and sharp waves. To distinguish the spike waves and sharp waves from the normal EEG waves, many signal processing methods were proposed in recent years. The study by Alharthi, M.K et al. focused on EEG seizure onset detection using a combination of the discrete wavelet transform (DWT) and a deep learning model consisting of a 1D-Convolutional Neural Network (CNN) with bidirectional long short-term memory (Bi-LSTM) [4]. They achieved the results of 96.87 % accuracy, 96.85 % sensitivity and 96.98 % precision in their experiment. Zarei, A et al. explored the use of orthogonal matching pursuit (OMP) combined with a support vector machine (SVM) classifier for detecting seizure onsets [5]. Their study reported a sensitivity of 96.81 % and a false positive (FP) rate of 2.74 %. In another study by Bhattacharyya, A. et al., the tunable-Q wavelet transform (TQWT) was proposed to decompose EEG epilepsy signals [6]. They then calculated entropy measures based on the decomposed signals. Their approach achieved an accuracy of 98.6 %. Li, C. et al. Proposed the use of common spatial pattern (CSP) to select relevant EEG channels for seizure onset detection [7]. They combined CSP with empirical mode decomposition (EMD) and an SVM model and obtained the result of a sensitivity of 97.34 % and a false positive rate of 2.5 %. In the research conducted by Oweis, R.J. et al., they utilized the Hilbert-Huang transform (HHT) in the frequency domain for seizure detection [8]. Their method achieved an accuracy of 94 % and specificity of 96 %. Hu, W. et al. highlighted the use of mean amplitude spectrum (MAP) combined with a CNN model for classifying the seizure active and seizure free data [9]. Their approach reported a classification accuracy of 86.25 %. Bomela, W. et al. developed a complex brain connection method for real-time seizure detection [10]. They supported the result of a sensitivity of 93.6 % and a false positive rate of 0.16 per hour in their study. Shayeste, H. et al. developed a short-time Fourier transform (STFT) algorithm based on the bagging technique and a decision tree model for automatic seizure detection [11]. Their approach received high accuracy, sensitivity, and specificity, with reported values of 99.56 %, 99.52 %, and 99.62 %, respectively. Amiri, M. et al. utilized Sparse CSP combined with an adaptive STFT-based synchro squeezing transform for automatic seizure detection [12]. Their method achieved a sensitivity of 98.44 %, specificity of 99.19 %, and accuracy of 98.81 %. In our previous work, DWT and RUSBoosted tree Ensemble methods were combined to detect EEG epilepsy seizure onset in real-time application, and achieved 96.15 % sensitivity, 96.38 % accuracy, 3.24 % FP rate and 10.42 s delay results [13]. Furthermore, TQWT and CNN model were also applied our seizure detection work, the results received 97.57 % in accuracy, 98.90 % in sensitivity, 2.13 % in FP rate and 10.46-s delay [14].

To address the robustness issue in EEG-based epilepsy detection, researchers have also developed machine learning and deep learning methods. Omidvar, M. et al. Proposed the use of a SVM model to classify EEG signals decomposed at the 5th level using the 5-db DWT [15]. They reported an accuracy of 98.7 % as the result in their paper. Donos, C. et al. employed the random forest algorithm to detect early seizures using intracranial EEG data [16]. Their method obtained a result of 93.84 % sensitivity. Gao, Y. et al. focused on deep learning and utilized a deep CNN to classify seizure activity in EEG data [17]. Their approach achieved an average classification accuracy of 90 % in epilepsy seizure detection. Cao, X. et al. used LSTM networks to directly detect seizure onset [18]. They provided the result with an accuracy of 96.3 % in their experiment. Wang, X et al. proposed a stacked 1D-CNN model for automatic seizure onset detection [19]. Their approach obtained an accuracy of 88.14 % and a false positive rate of 0.38 %.

Combining signal processing and image classification techniques using CNN models has shown promising results in EEG research. Chen, H et al. utilized mutual information (MI) algorithm to calculate brain graph data and combined it with a graph CNN model for detecting subjects with attention-deficit/hyperactivity disorder (ADHD) using EEG signals [20]. In their study, they received an accuracy of 94.67 % on the test data. Ozcan, A.R. et al. employed a 3D-CNN model to classify features extracted from EEG signals, including statistical parameters and band power spectrum, in the context of seizure prediction. Their method achieved a sensitivity of 85.7 % and a false positive rate of 0.096 per hour [21]. In our previous work, 3D-CNN classifier was proposed to classify the EEG alcoholic brain connectivity data and received the results of 96.25 ± 3.11 % accuracy [22]. Moreover, this kind of method also employed in our previous research [23]. Our 3D-CNN method provided the 97.74 ± 1.15 % accuracy, 96.91 ± 2.76 % sensitivity, and 98.53 ± 1.97 % results.

In this study, a bandpass filter using a 6th-order Butterworth zero-phase algorithm is applied to denoise the raw EEG data within the frequency range of 1–60 Hz. To extract features from the EEG signals, STFT spectrums provide a time-frequency representation of the data. The obtained spectrums were then transformed into graph data, which serves as the input for the Google-Net CNN models. To implement the approach in real-time, a sliding window technique with a duration of 1.35 s and a 1-s overlap is utilized. The experiments of this study were conducted on a Dell workstation equipped with an Intel I9–10900K CPU, 64 GB memory, and an Nvidia 2080ti GPU. MATLAB 2021b, along with the Deep Network Designer toolbox, was used for the deep learning work and model development.

In this paper, Section 1 briefly introduces the background and research problems. Section 2 describes the methodology which includes the signal processing, feature extraction and CNN model classification. Section 3 reports the results of the proposed method. Section 4 discusses the statistical analysis in time-frequency spectrum analysis, brain rhythms selection, and evaluation of different CNN models. Moreover, the previous works of the database CHB-MIT were also listed and evaluated in Section 4. Section 5 concludes the paper.

## 2 Material

The CHB-MIT database, collected by Boston Children's Hospital, consists of EEG data from 23 subjects [24]. The database included 5 males ranging in age from 3 to 22 years and 17 females ranging in age from 1.5 to 19 years. The EEG data in the CHB-MIT database was recorded using scalp EEG standard 10–20 system caps, with a sampling rate of 256 Hz. The data was collected from 22 bipolar channels. In addition, six specific electrodes P3–O1, FP2–F8, P8–O2, P7-T7, T7-FT9, and FT10-T8 were utilized in this experiment. These electrodes are strategically positioned closer to the frontal, temporal, and occipital regions, aligning with the seizure onset zones of the selected patients as outlined in Table 1. For this study, a subset of the CHB-MIT Database was selected, consisting of 16 patients. Patients who had seizures characterized by amplitude depression were excluded from the analysis. Upon closer examination of EEG signals for patients with high detection delay, it became apparent that seizures often initiated with amplitude depression before the onset of synchronization, characterized by high-amplitude oscillations [10]. In these cases, where the algorithm lacks the capability to detect amplitude depression, significant delays were observed. The details of the selected subjects can also be found in Table 1 of the study.

**Table 1:** Information about Database CHB-MIT of this study.

Case	Data collected (hour)	NS	Seizure duration (second)	Seizure type	Seizure onset zone
Patient 1	26	7	40,27,40,51,90,93,101	SP, CP	Temporal
Patient 2	17	3	82,81,9	SP, CP, GTC	Frontal
Patient 3	37	7	52,65,69,52,47,64,53	SP, CP	Temporal
Patient 4	24	4	49,111,102,116	SP, CP, GTC	Temporal, Occipital
Patient 5	16	5	115,110,96,120,117	CP, GTC	Frontal
Patient 7	29	3	86,96,143	SP, CP, GTC	Temporal
Patient 8	18	5	171,190,134,160,264	SP, CP, GTC	Temporal
Patient 9	32	4	64,79,71,62	CP, GTC	Frontal
Patient 10	22	7	35,70,65,58,76,89,54	SP, CP, GTC	Temporal
Patient 17	17	3	90,115,88	SP, CP, GTC	Temporal
Patient 18	17	6	50,30,68,55,68,46	SP, CP	Temporal, Occipital
Patient 19	16	3	78,77,81	SP, CP, GTC	Frontal
Patient 20	17	8	29,30,39,38,35,49,35,39	SP, CP, GTC	Temporal
Patient 22	17	3	58,74,72	Not reported	Temporal, Occipital
Patient 23	15	7	113,20,47,71,62,27,84	Not reported	Frontal
Patient 24	13	16	25,25,29,25,32,27,19,24,22,19,70,16,27,17,66,68	SP, CP	Temporal

Here, ‘NS’ is number of seizures, ‘SP’ is simple partial seizures, ‘CP’ is complex partial seizures, and ‘GTC’ is generalized tonic-clonic seizures.

## 3 Methodology

**Fig. 1:**
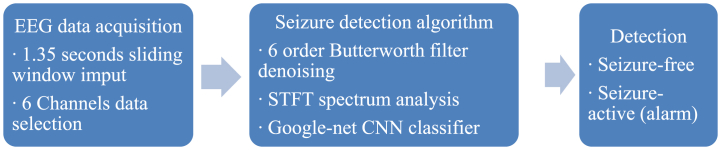
The framework of seizure detection via STFT spectrum analysis and Google-net CNN model.

Fig. 1 illustrates the progression of the EEG data acquisition, main seizure detection algorithm and generating alarms of detection. To reduce computational costs, six specific channels are selected for analysis. These channels include P3 – O1, FP2 – F8, P8 – O2, P7 - T7, T7 - FT9, and FT10 - T8. To enable real-time application, a sliding window technique is employed. The sliding window has a size of 1.35 s, which corresponds to 345 samples of EEG data. This approach allows for continuous analysis of the EEG signals by processing them in overlapping segments. The EEG raw data is subjected to band-pass filtering using a 6th-order Butterworth algorithm. This filtering process helps to remove unwanted noise and artifacts from the EEG signals, focusing on frequency components within the range of interest. Time-frequency analysis is performed on the filtered EEG data to extract specific frequency bands of interest. This analysis provides information on how the frequency content of the EEG signals changes over time, capturing transient characteristics such as those seen during seizure activity. The resulting time-frequency spectra are converted into a graph representation with dimensions of 120 × 344, which serves as the input for the Google-net CNN model.

### 3.1 Pre-processing

In this study, a 6th-order Butterworth algorithm is applied as a band-pass filter to the raw EEG data. The purpose of this filtering process is to selectively retain frequency components within the range of interest while attenuating frequencies outside this range. By applying the 6th-order Butterworth filter, frequencies outside the desired range from 1 to 60 Hz are attenuated, reducing the impact of noise and artifacts on the EEG signals. The filtered EEG signals primarily contain frequency components within the specified range, which is important for subsequent analysis and feature extraction steps in the proposed method.

### 3.2 Short-time fourier transform

In this paper, the STFT is utilized as a time-frequency analysis technique to decompose the EEG signal into different frequency sub-bands and components. Specifically, the STFT is used to construct the time-frequency domain spectrum of the EEG signal within the frequency range of 20–60 Hz. This frequency range is of interest in this study, because it contains relevant information related to the detection of epilepsy seizures. The STFT provides a representation of the signal in both the time and frequency domains by applying a series of Fourier transforms to overlapping segments of the signal. Based on the Fourier transform, STFT analysis considers the window function of time varying EEG fragments which are converted into frequency and time axes. The formula of Fourier transforms and STFT analysis are shown in equations (1), (2).

(1) $$\begin{array}{c} S\left(\omega \right)=\sum_{t}\bar{s}\left(t\right)e^{j\omega t}\; \end{array}$$

(2) $$\begin{array}{c} S\left(\omega \right)=\sum_{t}\bar{s}\left(t\right)g\left(t-u\right)e^{j\omega t} \end{array}$$

where ω is the selected frequency band, *g(t-u)* is the window function. Here, the window is selected as hamming 2 samples, and the number of overlapped samples is selected as 1. The input data from the *K* domains are donated by ${\bar{x}=\left[{\bar{x}}^{1},\ldots ,{\bar{x}}^{K}\right]}^{T}\in R^{K\times d}\text{,}$ where $\bar{x}\in R^{d\times 1}$, and the epoch with the time index *t* is given as $\bar{x}\left(t\right)$.

**Fig. 2:**
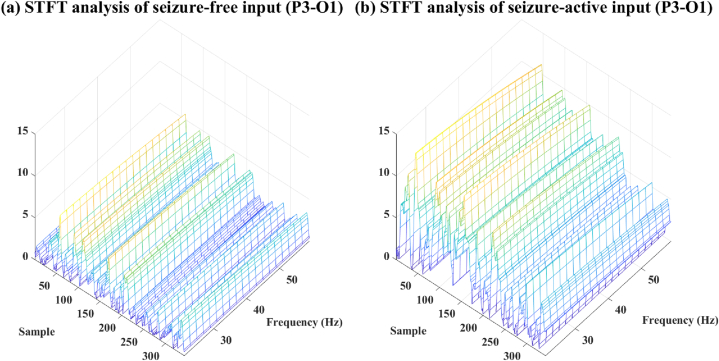
(a) STFT spectrum of seizure free data, (b) STFT spectrum of seizure active data. The seizure active data is collected from case *‘Chb01_03’* from 3009s to 3011s, and the seizure free data is collected in the same case from 2800s to 2802s.

The frame width used in the STFT is set to 128 samples. Consequently, the frequency resolution of the STFT spectrum power is 0:2:128, and the time resolution is 1:1:128. Additionally, the frequency resolution for the STFT is chosen as 2 Hz. After the STFT analysis, the one-channel EEG data is transformed into a 20 × 344 image-like dataset, as illustrated in Fig. 2, (a) is STFT spectrum of seizure free data and (b) is STFT spectrum of seizure active data. Following the extraction of STFT spectra for the six selected channels, the next step in the proposed approach is to merge them. The spectra from each channel are combined to form a single input matrix with a size of 120 × 344 per epoch.

### 3.3 Classification through deep learning method

**Fig. 3:**
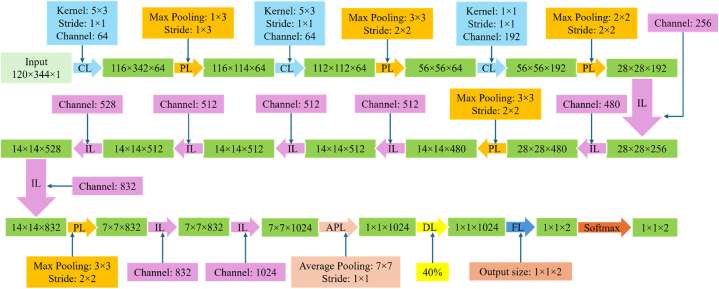
The 29-layer Google-net CNN architecture, ‘CL’ is convolution layer, ‘PL’ is max pooling layer, ‘IL’ is inception layer, ‘APL’ is average pooling layer, ‘DL’ is dropout layer and ‘FL’ is fully connected layer.

According to the size of the input data through STFT analysis, a 29-layer Google-Net CNN is constructed and shown in Fig. 3.

#### 3.3.1 Leaving one out training method

In the study, the leave-one-out training method is employed to evaluate the performance of the proposed method. This approach involves using one set of data as the test set while using the remaining data for training. In this experiment, a total of 16 models are trained, with each model being trained on a different combination of training and test data. For training purposes, the EEG raw data from 10 min before seizure onset and 5 min after seizure onset are used for each subject data.

#### 3.3.2 Google-net convolutional neural network

**Fig. 4:**
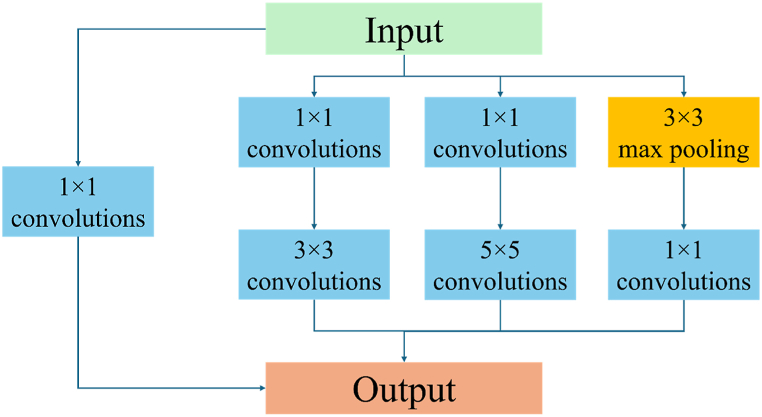
Architecture of an inception in Google-Net CNN model.

In the proposed Google-net CNN model, the graph data with dimensions of 120 × 344 matrices are used as input. The model is trained using a learning rate of 0.01 and 30 epochs. The Google-Net CNN model consists of three individual convolution layers and a total of fifty-four convolutions using nine inceptions. All convolutions in the model utilize rectified linear activation function ReLU along with batch normalization. The three individual convolution layers in the model have sixty-four filters each, with convolution kernels of size 5 × 3, 5 × 3, and 1 × 1, respectively. The nine inceptions in the model are designed similarly, and the detailed architecture of an inception is depicted in Fig. 4.

In addition, the Google-Net CNN architecture includes a total of six pooling layers. These pooling layers consist of five max pooling layers and one average pooling layer. Six pooling layers of this architecture are selected as 1 × 3, 3 × 3, 3 × 3, 3 × 3, 3 × 3, 7 × 7 size and 1 × 3, 2 × 2, 2 × 2, 2 × 2, 2 × 2, 1 × 1 stride, respectively. To alleviate the occurrence of overfitting in the CNN model, a 40 % dropout layer is designed in the architecture. Finally, the last two layers of the CNN model consist of a fully connected layer and a Softmax classifier layer, respectively. Table 2 in the paper provides a summary of the Google-net architecture, including the hyperparameter settings for each layer.

**Table 2:** The Google-Net CNN architecture used in EEG seizure detection.

Level	Layer	Input data size	Output data size	Hyperparameter settings
–	Image-data input	120 × 344 × 1		
1	Convolution layer	120 × 344 × 1	116 × 342 × 64	Kernel size: 5 × 3 Stride: 1 × 1 Channel: 64
2	Max Pooling layer	116 × 342 × 64	116 × 114 × 64	Pooling size: 1 × 3 Stride: 1 × 3
3	Convolution layer	116 × 114 × 64	112 × 112 × 64	Kernel size: 5 × 3 Stride: 1 × 1 Channel: 64
4	Max Pooling layer	112 × 112 × 64	56 × 56 × 64	Pooling size: 3 × 3 Stride: 2 × 2
5	Convolution layer	56 × 56 × 64	56 × 56 × 192	Kernel size: 1 × 1 Stride: 1 × 1 Channel: 192
6	Max Pooling layer	56 × 56 × 192	28 × 28 × 192	Pooling size: 2 × 2 Stride: 2 × 2
7	Inception layer	28 × 28 × 192	28 × 28 × 256	Channel: 256
9	Inception layer	28 × 28 × 256	28 × 28 × 480	Channel: 480
11	Max Pooling layer	28 × 28 × 480	14 × 14 × 480	Pooling size: 3 × 3 Stride: 2 × 2
12	Inception layer	14 × 14 × 480	14 × 14 × 512	Channel: 512
14	Inception layer	14 × 14 × 512	14 × 14 × 512	Channel: 512
16	Inception layer	14 × 14 × 512	14 × 14 × 512	Channel: 512
18	Inception layer	14 × 14 × 512	14 × 14 × 528	Channel: 528
20	Inception layer	14 × 14 × 528	14 × 14 × 832	Channel: 832
22	Max Pooling layer	14 × 14 × 832	7 × 7 × 832	Pooling size: 3 × 3 Stride: 2 × 2
23	Inception layer	7 × 7 × 832	7 × 7 × 832	Channel: 832
25	Inception layer	7 × 7 × 832	7 × 7 × 1024	Channel: 1024
27	Average Pooling layer	7 × 7 × 1024	1 × 1 × 1024	Pooling size: 7 × 7 Stride: 1 × 1
28	Dropout layer	1 × 1 × 1024	1 × 1 × 1024	40 %
29	Fully Connected layer	1 × 1 × 1024	1 × 1 × 2	
–	Softmax	1 × 1 × 2	1 × 1 × 2	

A hold-out validation method is employed to validate the deep learning model during the training process. The training data is randomly divided into a training set and a validation set, with the validation set comprising 20 % of the training data. During training, the performance of each model is evaluated periodically using the validation set. The validation accuracy is calculated and recorded at regular intervals, with a validation frequency of 50 iterations. Table 3 presents the validation accuracy results obtained during the training process, showcasing the performance of each model on the validation set at different stages of training.

**Table 3:** The validation accuracy of STFT spectrum with Google-net training models.

Case	Validation Accuracy of STFT (%)
Patient 1	96.75
Patient 2	96.90
Patient 3	96.48
Patient 4	96.90
Patient 5	96.89
Patient 7	96.96
Patient 8	97.06
Patient 9	97.22
Patient 10	96.69
Patient 17	96.92
Patient 18	97.00
Patient 19	97.00
Patient 20	97.02
Patient 22	96.84
Patient 23	96.82
Patient 24	96.33
Mean ± SD	96.86 ± 0.22

**Fig. 5:**
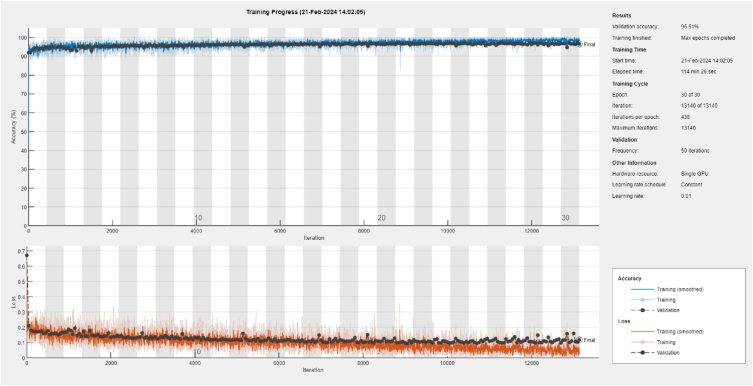
The training progress for *‘Patient 1′* case via STFT analysis and Google-net CNN based on MATLAB 2021b software.

Fig. 5 describes the progress of training the Google-net CNN model using STFT analysis for the *‘Patient 1'*. The figure shows the changes in loss and accuracy during the training process for both the training data and validation data. The training loss and accuracy curves indicate how well the model is fitting the training data over iterations. The validation accuracy curve shows the performance of the model on the validation set during training. According to the figure, the proposed Google-net CNN model successfully distinguished the imbalanced features of seizure-free and seizure-active states in this case. The model achieved a validation accuracy of 96.75 % for this particular case, indicating its ability to accurately classify EEG signals related to seizures.

## 4 Results and comparison

To evaluate the proposed real-time seizure onset detection method, four main parameters are applied in this study which include the accuracy, sensitivity, FP rate and the delay of the seizure onsets.

Accuracy measures how well the proposed method correctly identifies seizure onset events and non-seizure events, and the formula is described in equation (3)

(3) $$\begin{array}{c} Acc=\frac{TP+TN}{TP+TN+FP+FN} \end{array}$$

where ‘*TP*’, *‘TN’*, *‘FP’*, *‘FN’* correspond to the true positive, true negative, false positive and false negative.

Sensitivity, also known as recall or true positive rate, measures the ability of the algorithm to correctly identify seizure events or seizure onset. In active seizure detection, the goal is to accurately detect the occurrence of seizure activity in real-time EEG signals. Sensitivity quantifies the proportion of actual seizure events that are correctly detected by the algorithm. The algorithm of sensitivity is defined in equation (4)

(4) $$\begin{array}{c} Sen=\frac{TP}{Number\;of\;seizures} \end{array}$$

**Fig. 6:**
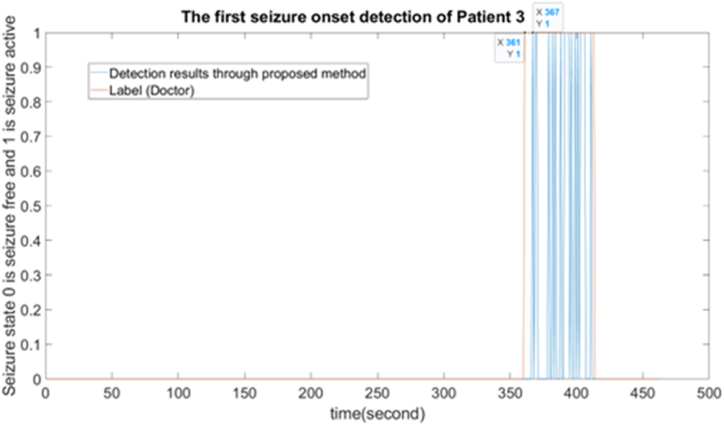
The first seizure onset detection of the Patient 3 through the proposed method.

The ‘delay’ of seizure onset refers to the temporal gap between the actual commencement of a seizure and its detection by the employed method. It gauges the precision of the detection algorithm in terms of time. Typically, this ‘delay’ is computed by measuring the time interval from the initiation of the seizure to the identification of its onset. The detail of ‘delay’ in the Patient 3 the first seizure onset is shown in Fig. 6 as follows.

Here, the doctor marked this onset from 361 s to 413 s, and our proposed method can detect this seizure beginning at 367 s. Thus, the ‘delay’ of this seizure is 6 s.

### 4.1 Results of the proposed method

In the real-time application based on Database CHB-MIT, 41,280 eigenvalues from 120 × 344 matrix graph data are selected. Google-net CNN model is utilized to evaluate the model using leaving one training method. As a result, Table 4 reported 97.74 % in accuracy, 98.90 % in sensitivity, 1.94 % in false positive rate, 9.85 s delay.

**Table 4:** Real time detection using STFT and Google-net method.

Case	Number of seizures	True positive	FP rate (%)	Sensitivity (%)	Delay (s)	Accuracy (%)
Patient 1	7	7	2.34	100.00	2.45	97.46
Patient 2	3	3	2.30	100.00	6.35	97.64
Patient 3	7	7	0.37	100.00	3.73	99.26
Patient 4	4	4	0.72	100.00	22.02	99.06
Patient 5	5	5	2.22	100.00	8.82	97.63
Patient 7	3	3	3.10	100.00	3.02	96.85
Patient 8	5	5	2.12	100.00	6.62	96.83
Patient 9	4	4	7.69	100.00	1.52	92.30
Patient 10	7	7	5.82	100.00	1.45	94.07
Patient 17	3	3	0.14	100.00	31.02	99.48
Patient 18	6	5	0.30	83.33	17.02	99.25
Patient 19	3	3	0.26	100.00	5.35	99.58
Patient 20	8	8	0.25	100.00	17.90	99.28
Patient 22	3	3	0.69	100.00	9.02	99.20
Patient 23	7	7	0.75	100.00	13.31	98.47
Patient 24	16	16	1.93	100.00	8.02	97.55
Total	91	90				
Mean			1.94		9.85	97.74
Sensitivity		98.90				

### 4.2 Comparison with different frequency bands

Determining which specific frequency range or band yielded the most promising results for real-time epilepsy seizure detection helps to reduce computing costs. Six frequency bands are considered in this experiment which include δ band (1–4 Hz), θ band (4–8 Hz), α band (8–12 Hz), β band (12–30 Hz), γ band (30–60 Hz) and selected frequency band (20–60 Hz), the comparison is described in Table 5.

**Table 5:** The comparison between different frequency band.

Frequency band	Accuracy (%)	Sensitivity (%)	FP rate (%)
δ band (1–4 Hz)	90.23	91.20	10.84
θ band (4–8 Hz)	92.45	93.41	8.93
α band (8–12 Hz)	93.38	93.41	7.61
β band (12–30 Hz)	94.56	96.70	5.94
γ band (30–60 Hz)	96.93	98.90	3.12
**Selected band (20–60 Hz)**	**97.74**	**98.90**	**1.94**

According to Table 6, the selected frequency band brain has been verified as the best frequency band to detect the EEG epilepsy seizure signal.

**Table 6:** Results of 2 Deep learning methods and proposed methods.

Deep learning methods	Accuracy (%)	Sensitivity (%)	FP rate (%)	Delay (s)
VGG-net CNN	95.89	84.62	3.70	8.33
Squeeze-net CNN	97.23	91.20	2.37	12.41
**Google-net CNN**	97.74	98.90	1.94	9.85

### 4.3 Comparison with different deep learning models

Two CNN methods are compared with the proposed Google-net CNN model in testing data which contains VGG-net CNN and Squeeze-net CNN. In these comparison, the same input data with the same validation method are applied to conduct the EEG epilepsy signal detection and compared with the results of the proposed method. The input data for the Google-Net CNN model was a 120 × 344 matrix of imaged-like data, while for VGG-net CNN and Squeeze-net CNN models, the first three layers of the Google-Net CNN model were used to represent a 224 × 224 × 3 input. The results of these three deep learning methods are summarized in Table 6.

Table 7 indicates that the Google-Net CNN model outperformed the VGG-net CNN and Squeeze-net CNN models in real-time EEG epilepsy seizure onset detection. The Google-Net CNN model, with its complex architecture and multiple layers, seems to have demonstrated better capabilities in capturing the relevant patterns and features in the EEG data for seizure detection.

**Table 7:** Real time detection using CWT and Google-net method.

Case	Number of seizures	True positive	FP rate (%)	Sensitivity (%)	Delay (s)	Accuracy (%)
Patient 1	7	7	3.02	100.00	16.88	96.59
Patient 2	3	3	2.67	100.00	5.69	97.28
Patient 3	7	7	1.63	100.00	15.16	97.89
Patient 4	4	4	1.60	100.00	26.52	98.12
Patient 5	5	5	3.68	100.00	3.42	96.29
Patient 7	3	3	1.56	100.00	3.02	98.33
Patient 8	5	5	1.85	100.00	5.22	96.93
**Patient 9**	**4**	**4**	**53.08**	**100.00**	**−8.98**	**46.91**
Patient 10	7	7	6.09	100.00	3.16	93.70
Patient 17	3	3	2.01	100.00	15.02	97.63
Patient 18	6	5	0.72	83.33	25.62	98.79
Patient 19	3	3	0.84	100.00	2.35	98.91
Patient 20	8	8	3.38	100.00	14.52	96.18
Patient 22	3	3	3.33	100.00	6.35	96.42
Patient 23	7	7	8.31	100.00	6.59	90.94
Patient 24	16	16	9.16	100.00	5.15	90.28
Total	91	90				
Mean			6.43		9.11	93.20
Sensitivity		98.90				

## 5 Discussion

### 5.1 Time-frequency domain analysis

**Fig. 7:**
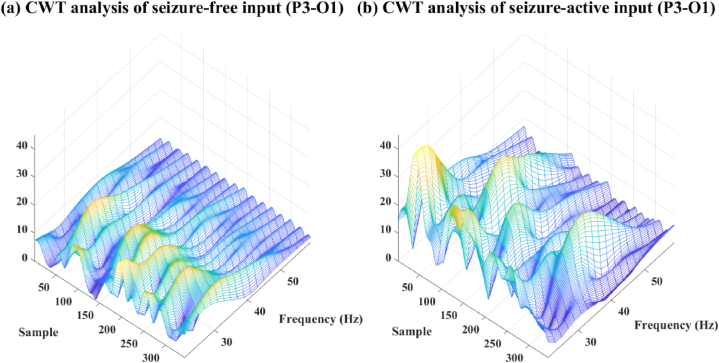
(a) CWT spectrum of seizure free data, (b) CWT spectrum of seizure active data. The seizure active data is collected from case *‘Chb01_03’* from 3009s to 3011s, and the seizure free data is collected in the same case from 2800s to 2802s.

Continuous wavelet transform (CWT) provides variable time and frequency resolution. It uses a wavelet function that can be adjusted in scale to analyse the signal at different frequencies with varying time resolution. Additionally, CWT can capture both high and low frequencies with good temporal localization. It can provide detailed information about transient events or signals with varying frequency content over time. Moreover, CWT is more flexible in terms of the choice of wavelet function and the ability to adapt the analysis to different frequency bands or signal characteristics. This allows for better customization and optimization based on the specific requirements of the application. In this study, the CWT spectrum also tests in the EEG seizure onset detection work, and the frequency resolution are selected into 2 Hz as well. The details of CWT spectrum of seizure free and seizure active states are shown in Fig. 7 (a) and (b), and the results are described in Table 7.

It is evident that, from Table 7, the CWT spectrum with Google-net CNN model is hard to detect case *‘Patient 09’,* which just achieved 46.91 % accuracy and 53.08 % FP rate. The STFT can provide a better performance in this research area.

### 5.2 Real-time application

In real-time applications, it is essential to process the data within a limited time frame to provide timely and actionable results. If the calculation time exceeds the overlapping time between consecutive windows, it can result in a delay that renders the detection impractical for real-time use. Therefore, selecting an appropriate window size is crucial to ensure the computational requirements of the method align with the desired real-time performance. Moreover, the parameter of delay is an important consideration when detecting EEG seizure onset. A large sliding window input, due to a larger window size, can result in a significant delay parameter. This delay refers to the time it takes for the detection algorithm to identify the onset of a seizure after it occurs. A large delay can reduce the clinical relevance of the detection method, as timely intervention or response may be compromised. In this study, a 1.35-s sliding window is selected to balance the detection and avoid significant delays in real-time applications.

In our previous work [13,14], the eigenvalue calculation step increased the overall calculation time. The decision to utilize the STFT spectrum directly as input for the Google-Net CNN model has proven to be an effective strategy for minimizing processing time and enabling real-time applications in EEG seizure detection. The processing time of the proposed method is just 0.02 s.

### 5.3 Previous works comparison

Comparisons results with the related works in EEG epilepsy seizure onset detection are listed in Table 8. The proposed method receives 97.74 % in accuracy, 98.90 % in sensitivity, 1.94 % in false positive rate, and 9.85-s results in the testing data. Compared with the previous related work, the proposed method can achieve satisfactory detection results using the CHB-MIT database. Moreover, the study highlights the efficiency of the proposed method in terms of processing time. It reports a processing time of just 0.02 s for every 1.35-s EEG episode. This indicates that the method is computationally efficient and capable of performing real-time seizure detection with minimal delay.

**Table 8:** Comparison of the related works in EEG epilepsy seizure detection.

Reference	Techniques	Sen (%)	Acc (%)	FP rate (%)	Delay (s)
Zarei, A. et al. (2021) [5]	DWT + SVM	96.81	97.09	2.74	–
Abdelhameed, A. et al. (2021) [25]	LSTM	98.72	98.79	1.14	–
Alharthi, M.K. et al. (2022) [4]	DWT + LSTM	96.85	96.87	3.02	–
Amiri, M. et al. (2022) [12]	STFT + SVM	98.44	98.81	0.81	–
Shayeste, H. et al. (2022) [11]	STFT + Decision Tree	99.52	99.56	0.38	–
L. Jiang. et al. (2022) [26]	Functional brain network + SVM	97.72	96.67	4.38	–
Liu, S. et al. (2023) [27]	Power spectrum density + SVM	99.90	99.95	0.01	–
Our previous work (2022) [13]	DWT + RUS Boosted	96.15	97.00	3.24	10.42
Our previous work (2023) [14]	TQWT + CNN	98.90	97.57	2.13	10.46
**Proposed method**	STFT + Google-net CNN	98.90	97.74	1.94	9.85

However, the proposed method currently cannot detect seizures characterized by amplitude depression. Addressing this limitation represents a key area for future research in EEG seizure onset. The experiment conducted in the study had a frequency resolution of 2 Hz instead of 1 Hz. This reduction in frequency resolution was due to limitations in the CPU memory capacity of the workstation used for the study. A higher frequency resolution could potentially provide more detailed information and improve the accuracy of the seizure detection. The selection of CNN models was limited in the study due to GPU memory constraints. As a result, the study could not include CNN models such as Efficient-Net CNN and ResNet-50 CNN. These models are known for their effectiveness in various computer vision tasks and may have provided additional insights and potentially improved the performance of the proposed method if they could have been utilized.

## 6 Conclusion

This study proposes an EEG-based real-time epilepsy seizure detection approach that combines signal processing techniques with deep learning methods, specifically utilizing time-frequency spectrum and Google-Net CNN models. This approach starts by applying the STFT method to extract signal features and remove redundant information. This helps to improve the robustness of epilepsy detection using EEG signals. The study then employs the Google-Net CNN model, designed specifically for image-like data, and compares its performance with the Squeeze-net and VGG-net CNN models. The evaluation results demonstrate that the Google-Net CNN model achieves better performance in classifying the image-like data. Additionally, the STFT method is found to be superior to the CWT in terms of reducing the false positive rate. The proposed real-time seizure detection method achieved impressive results on the CHB-MIT Database, with 97.74 % accuracy, 98.90 % sensitivity, 1.94 % false positive rate, and 9.85-s delay when utilizing the STFT spectrum. Based on these findings, the study concludes that the proposed method is suitable for real-time seizure detection and holds great potential for impactful clinical applications. The future work includes testing the seizure prediction aspect of the method in clinical applications using portable EEG devices and brainwave monitors and realize detect seizures characterized by amplitude depression.

## Data availability statement

The data used in this manuscript is a publica data (CHB-MIT database), which can download in https://physionet.org/content/chbmit/1.0.0/.

## Funding

None.

## Ethical approval

Not required.

## CRediT authorship contribution statement

**Mingkan Shen:** Writing – review & editing, Writing – original draft, Visualization, Validation, Supervision, Software, Resources, Project administration, Methodology, Investigation, Formal analysis, Data curation, Conceptualization. **Fuwen Yang:** Supervision. **Peng Wen:** Writing – review & editing, Supervision. **Bo Song:** Writing – review & editing, Writing – original draft, Supervision. **Yan Li:** Supervision.

## Declaration of competing interest

The authors declare that they have no known competing financial interests or personal relationships that could have appeared to influence the work reported in this paper.
